# Fish growth enhances microbial sulfur cycling in aquaculture pond sediments

**DOI:** 10.1111/1751-7915.13622

**Published:** 2020-07-06

**Authors:** Keke Zhang, Xiafei Zheng, Zhili He, Tony Yang, Longfei Shu, Fanshu Xiao, Yongjie Wu, Binhao Wang, Zhou Li, Pubo Chen, Qingyun Yan

**Affiliations:** ^1^ Southern Marine Science and Engineering Guangdong Laboratory (Zhuhai) School of Environmental Science and Engineering Environmental Microbiomics Research Center Sun Yat‐sen University Guangzhou Guangdong 510006 China; ^2^ College of Agronomy Hunan Agricultural University Changsha 410128 China; ^3^ Swift Current Research and Development Centre Agriculture & Agri‐Food Canada Swift Current SK Canada

## Abstract

Microbial sulfate reduction and sulfur oxidation are vital processes to enhance organic matter degradation in sediments. However, the diversity and composition of sulfate‐reducing bacteria (SRB) and sulfur‐oxidizing bacteria (SOB) and their environmental driving factors are still poorly understood in aquaculture ponds, which received mounting of organic matter. In this study, bacterial communities, SRB and SOB from sediments of aquaculture ponds with different sizes of grass carp (*Ctenopharyngodon idellus*) were analysed using high‐throughput sequencing and quantitative real‐time PCR (qPCR). The results indicated that microbial communities in aquaculture pond sediments of large juvenile fish showed the highest richness and abundance of SRB and SOB, potentially further enhancing microbial sulfur cycling. Specifically, SRB were dominated by *Desulfobulbus* and *Desulfovibrio*, whereas SOB were dominated by *Dechloromonas* and *Leptothrix*. Although large juvenile fish ponds had relatively lower concentrations of sulfur compounds (i.e. total sulfur, acid‐volatile sulfide and elemental sulfur) than those of larval fish ponds, more abundant SRB and SOB were found in the large juvenile fish ponds. Further redundancy analysis (RDA) and linear regression indicated that sulfur compounds and sediment suspension are the major environmental factors shaping the abundance and community structure of SRB and SOB in aquaculture pond sediments. Findings of this study expand our current understanding of microbial driving sulfur cycling in aquaculture ecosystems and also provide novel insights for ecological and green aquaculture managements.

## Graphical abstract

In aquaculture pond sediments, sulfate‐reducing bacteria (SRB) produced AVS through sulfate reduction, and sulfur‐oxidizing bacteria was especially important to reduce AVS. High concentrations of sulfur compounds (TS, AVS, and ES) could inhibit SRB and sulfate reduction. Disturbance to sediments by fish activities could enhance the sulfate reduction and sulfur oxidation in sediments.

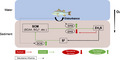

## Introduction

As a key part of global biogeochemical cycles, sulfur cycling mediated by sulfate reduction and sulfur oxidation plays a critical role in nutrient metabolism processes (Li *et al*., 2018). Sulfur compounds are important oxidants or reductants for microbial respiration in sediments, which are mainly catalysed by SRB and SOB (Santander‐De Leon *et al*., 2013). On the one hand, dissimilatory sulfate reduction mediated by SRB is the dominant anaerobic mineralization pathway of organic matter degradation in sediments (Watanabe *et al*., 2013), which consequently release hydrogen sulfide simultaneously into ecosystems (Tian *et al*., 2017). On the other hand, sulfur oxidation mediated by SOB can convert hydrogen sulfide to elemental sulfur or sulfate, and remove toxic and foul‐smelling compounds such as H_2_S (Cheng *et al*., 2018). Generally, high sulfate concentrations and organic matter deposition in marine sediments enhance sulfate reduction, releasing sulfide into pore water (Kondo *et al*., 2004; Aller, 2014). However, high oxidation efficiency of sulfide could restrict translocation of dissolved sulfide into water–sediment interfaces (Dyksma *et al*., 2016), and limited oxidants in sediments may lead to a formation of various intermediate sulfur compounds (e.g. thiosulfate and elemental sulfur) (Van Den Ende and Gemerden, 2010). The importance of sulfur cycling in sediments has been increasingly recognized (Choi *et al*., 2006), but studies of microbial sulfur cycling mainly focused on marine (Böttcher, 2001) and freshwater environments (Bryukhanov *et al*., 2018). However, little is known about high‐density aquaculture ecosystems in coastal areas, which has been quickly developed in the past decade (FAO, 2018).

Coastal aquaculture has high‐nutrient loads and low dissolved oxygen situation (Sachidanandamurthy and Yajurvedi, 2006). A low efficiency of feed utilization in aquaculture ponds leads to a great accumulation of organic matters such as uneaten formulated feeds and fish faeces in sediments (Holmer and Kristensen, 1996; Asami *et al*., 2005). Ultimately, these accumulative organic matters will be degraded by SRB, as the sulfate reduction is a major driver in transforming organic carbon to CO_2_ (Anantharaman *et al*., 2018). Moreover, sulfate usually is the most abundant water‐soluble electron acceptor for SRB (Huycke and Gaskins, 2004; Knossow *et al*., 2015). This is especially true in ponds located at estuarine areas along coastline of South China. SRB in aquaculture pond sediments also can use other electron donors such as short chain fatty acids (SCFA), which are abundant in formulated feed (Hansen and Blackburn, 1995). The degradation and mineralization of these organic matters can lead to serious deterioration of water quality, resulting in eutrophication of aquaculture ponds (Krishnani *et al*., 2010b). Furthermore, sulfur compounds produced by SRB can be oxidized to sulfate by SOB (Barton *et al*., 2014; Ihara *et al*., 2017) and therefore maintain sulfide concentrations at a safe level (Fernandes *et al*., 2010). Although sulfur cycling plays such important roles in nutrient cycling and environmental safety in aquaculture ecosystems, and current research mainly focused on the composition of SRB and SOB (Rubio‐Portillo *et al*., 2019). However, the regulatory mechanisms of SRB and SOB remain poorly understood in aquaculture ponds.

Sulfur cycling could be affected by various factors in aquaculture pond sediments (Duc *et al*., 2018). Generally, amounts of feed supply and feed utilization efficiency are the major factors that influence fish growth (Biswas *et al*., 2005; Kondo *et al*., 2012). Therefore, excessive nutrient input is the most common strategy in the aquaculture industry to increase production, which also will significantly affect compositions and bio‐functions of bacterial communities in sediments (Leflaive *et al*., 2008). Besides, suspended sediments due to activities of aquatic animals not only increase turbidity in water column (Croel and Kneitel, 2011), but also disturb microbial communities in sediments (Mendoza‐Lera and Mutz, 2013). In aquaculture ponds, larger fish usually have strong disturbances to water–sediment interfaces, which will further influence the bacterial communities in sediments (Jochum *et al*., 2017). Combined with field observations, a previous study found that fish size could impact the accumulation and consumption of sulfur‐containing substances (Barnes and Jennings, 2007).

In this study, we aimed to understand how feeding practices and fish sizes will affect sulfur‐cycling microbial communities in the sediment of aquaculture ponds. We hypothesized that sulfur‐cycling microbial communities would differ among aquaculture ponds with different sizes of fish, and nutrient accumulation and sediment suspension due to fish activities would enhance the sulfur‐cycling microbial communities in the sediment. To test these hypotheses, we examined sulfur‐cycling microbial communities in aquaculture ponds cultured with three sizes of grass carp by high‐throughput sequencing and qPCR of 16S rRNA (Griffiths *et al*., 2000), *dsrB* and *soxB* (Zhang *et al*., 2017) genes. We found that aquaculture ponds with large grass carp showed more abundant sulfur‐cycling microorganisms than those with smaller sizes of fish, and sulfur compounds and sediment suspension were the major driving factors. This study provides novel knowledge that strong fish activities (bioturbation) decreasing toxic sulfur compounds, changing sulfur‐cycling microbial communities in aquaculture pond sediments.

## Results

### Environmental parameters in grass carp aquaculture ponds

Our results showed clear differences about physicochemical parameters of sediment and water in aquaculture ponds with different sizes of fish (Table 1). With the increase in fish sizes, total sulfur (TS), elemental sulfur (ES) and acid‐volatile sulfur (AVS) in the sediment all significantly (*P < *0.05) decreased. However, total organic carbon (TOC) and total nitrogen (TN) in the sediment were not significantly influenced by fish size. Total suspended solids (TSS) in the water were significantly (*P < *0.05) higher in large juvenile fish ponds than larval and small juvenile fish ponds. By contrast, the transparency was significantly (*P < *0.05) lower in large juvenile fish ponds. TN and nitrate in the water were also significantly (*P < *0.05) increased with the increase in fish sizes.

**Table 1:** Summary of sediment and water physicochemical properties in aquaculture ponds with different sizes of grass carp.

Habitat	Parameter	Ponds		
		L	SJ	LJ
Sediment	pH	7.66 ± 0.52	7.49 ± 0.43	7.41 ± 0.33
	AVS (mg g^−1^)	0.44 ± 0.19^a^	0.40 ± 0.19^a^	0.11 ± 0.05^b^
	TS (mg g^−1^)	4.10 ± 1.34^a^	2.45 ± 1.58^b^	1.16 ± 0.25^c^
	ES (mg g^−1^)	0.63 ± 0.24 ^a^	0.43 ± 0.11^ab^	0.25 ± 0.16^b^
	Sulfate (mM l^−1^)	2.28 ± 0.96	2.77 ± 1.04	2.27 ± 0.34
	TOC (mg g^−1^)	20.85 ± 3.48	18.06 ± 7.06	19.75 ± 3.38
	TN (mg g^−1^)	1.89 ± 0.44	1.63 ± 0.79	1.58 ± 0.29
Water	TSS (mg l^−1^)	2.47 ± 0.75^b^	2.72 ± 1.01^b^	4.05 ± 0.17^a^
	Transparency (m)	0.22 ± 0.08^a^	0.18 ± 0.05^a^	0.12 ± 0.02^b^
	TN (mg l^−1^)	5.48 ± 1.15^c^	7.01 ± 1.84^b^	8.72 ± 0.62^a^
	Nitrate (mg l^−1^)	0.19 ± 0.12^c^	1.64 ± 1.60^b^	3.55 ± 0.86^a^

Data showed as Mean ± SD, *n* = 12. AVS, acid‐volatile sulfur; ES, elemental sulfur; L, larval; LJ, large juvenile; SJ, small juvenile; TN, total nitrogen; TOC, total organic carbon; TS, total sulfur; TSS, total suspended solids. Values with different superscript letters mean significantly different (*P* < 0.05) among ponds, and same letters or without letters indicate no significant difference.

### Fish growth increased bacterial diversity in aquaculture pond sediments

**Fig. 1:**
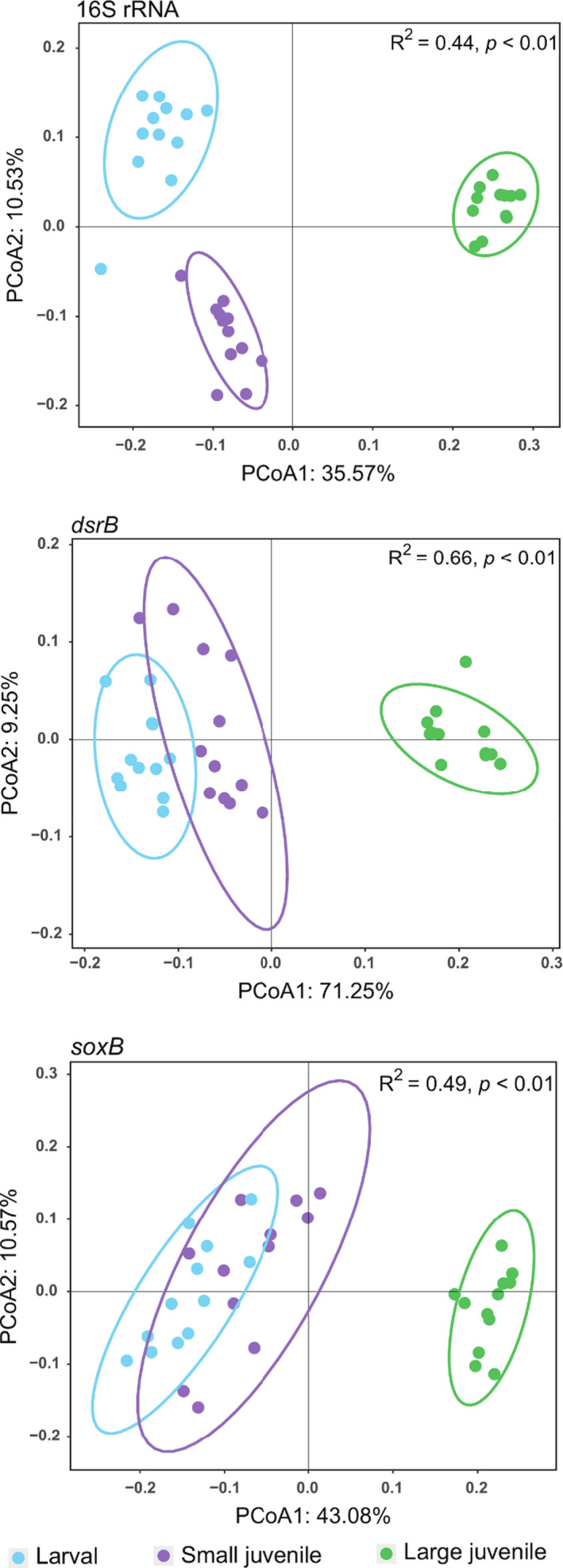
Principal coordination analysis (PCoA) showing the dissimilarity of microbial communities according to sequencing results of 16S rRNA, *dsrB* and *soxB* genes. Anosim test was employed to indicate the significance of dissimilarity.

**Fig. 2:**
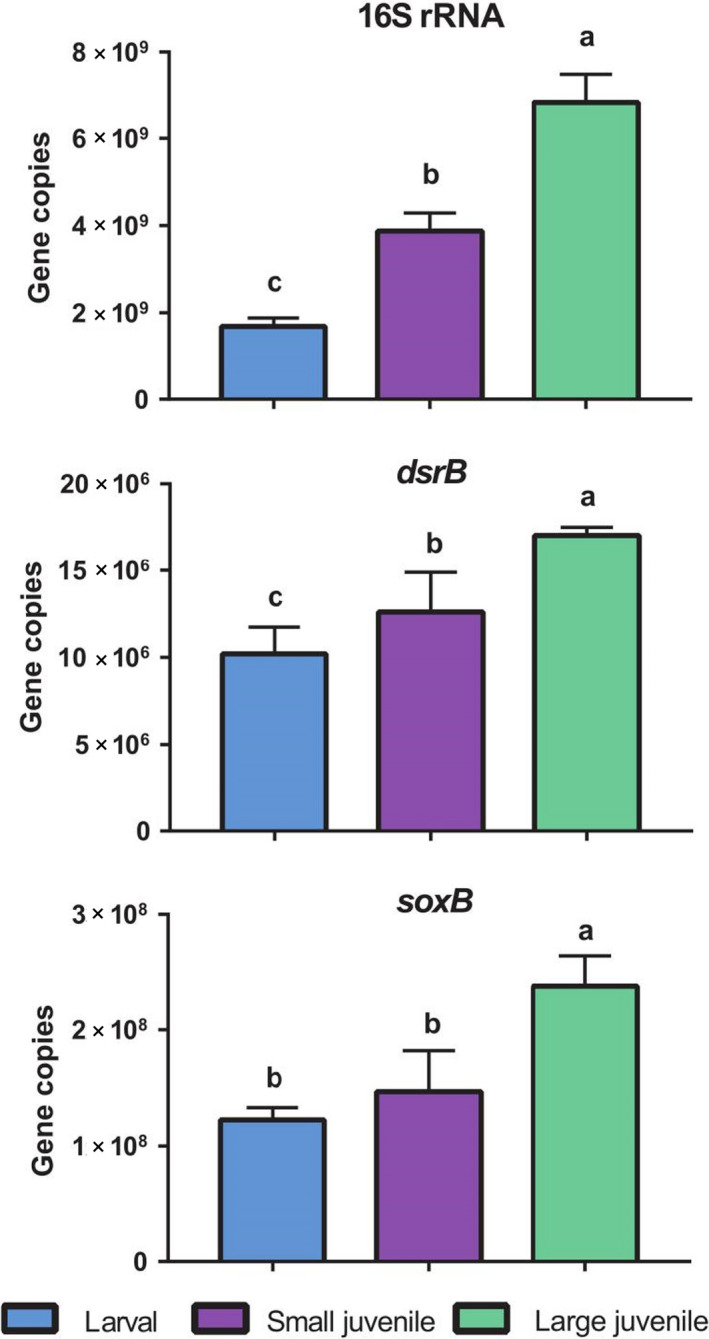
Abundances of 16S rRNA, *dsrB* and *soxB* genes (copies per g wet sediment) in ponds cultured with larval, small juvenile and large juvenile grass carp. Data presented as mean ± SD (*n* = 12)

Our high‐throughput sequencing and qPCR analysis were used to examine changes in bacterial diversity and abundance among different fish ponds. In particular, the total number of OTUs obtained from 16S rRNA, *drsB* and *soxB* genes was 14 926, 518 and 483 respectively. The large juvenile fish ponds showed significantly (*P < *0.05) higher number of OTUs and Shannon indices for bacterial community and SOB compared to larval and small juvenile fish ponds. However, for the SRB, Shannon indices had no significantly difference among larval, small juvenile and large juvenile fish ponds (Fig. S1). The bacterial community structure, as well as SRB and SOB composition in large juvenile fish ponds, was quite different from those in larval and small juvenile fish ponds visualized by the PCoA (Fig. 1). The qPCR analysis showed that the abundance of 16S rRNA, *dsrB* and *soxB* genes in large juvenile fish ponds was the highest (Fig. 2). Specifically, the abundance of *dsrB* gene in the large juvenile fish ponds accounted for 0.25% of the 16S rRNA gene abundance, while the *soxB* gene accounted for 3.5% of total bacterial abundance in large juvenile fish ponds.

### The composition of bacterial communities, SRB and SOB in aquaculture pond sediments

To understand dominant sulfur‐cycling microorganisms and compare their relative abundance among aquaculture ponds with different sizes of fish, we filtered unidentified OTUs at the genus level and focused on the classified top 10 genera (Fig. S2). In the 16S rRNA gene data set, *Geobacter* and *Anaerolinea* were dominant genera in all samples. *Anaerolinea, Desulfatiglans* and *Geobacter* were dominant genera in large juvenile fish ponds, while *Candidatus* Anammoximicrobium, *Desulfobacca*, *Pirellula* and *Coxiella* were more abundant in larval and small juvenile fish ponds than those in large juvenile fish ponds (*P* < 0.05). For SRB, *Desulfobulbus* and *Desulfovibrio* were dominant genera in all samples, and *Desulfovibrio, Desulfomoniel, Desolfococcus* and *Desulfobulbus* were more abundant in small juvenile and large juvenile fish ponds than those in larval fish ponds (*P* < 0.05). For SOB, *Dechloromonas* and *Leptothrix* were dominant genera in all samples, and *Dechloromonas* was more abundant in larval and small juvenile fish ponds, while *Thiobacillus* showed higher abundance in large juvenile fish ponds (*P* < 0.05).

**Fig. 3:**
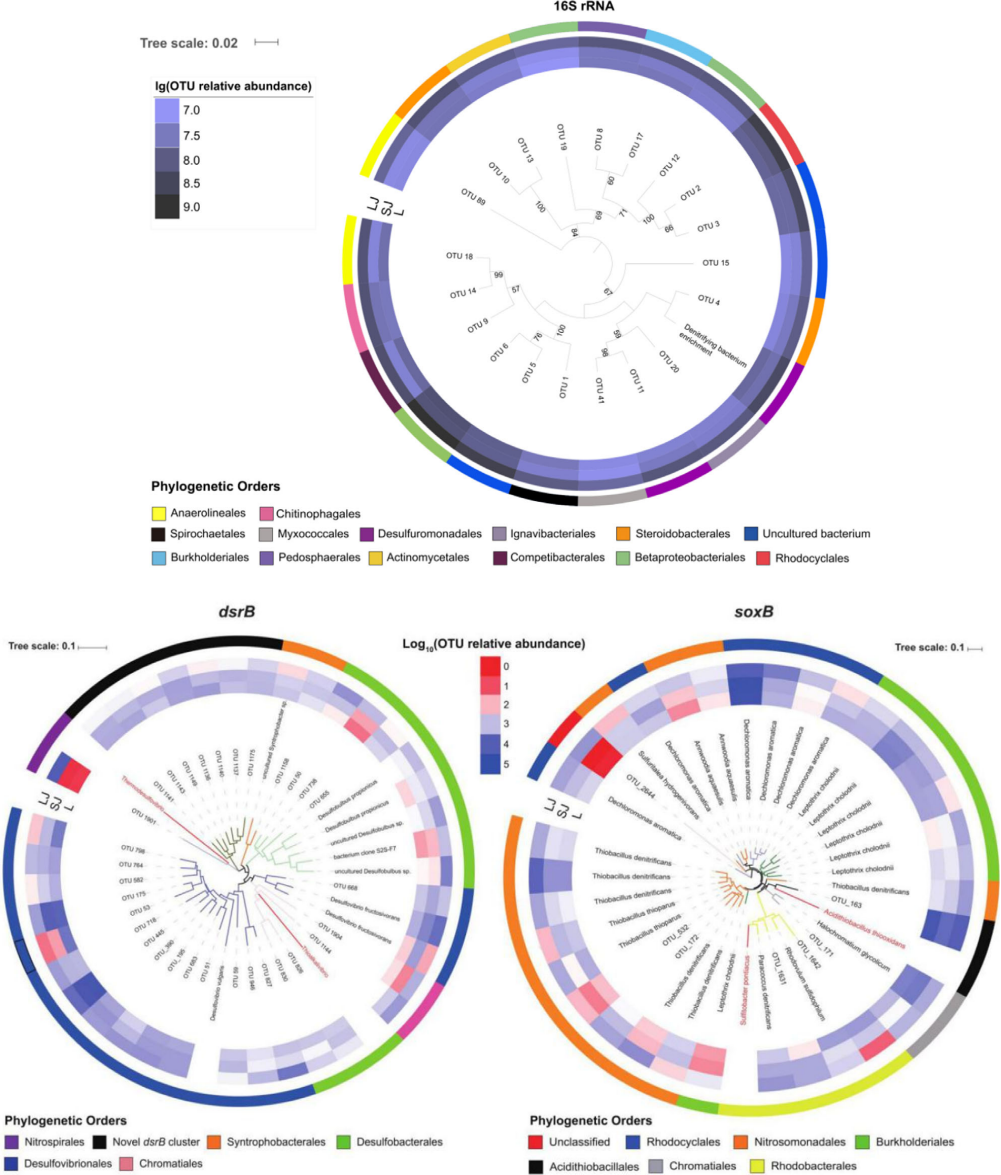
Neighbour‐joining phylogenetic trees generated according to the 16S rRNA, *dsrB* and *soxB* genes. The trees were constructed based on the inferred amino acids of representative nucleotide sequences of OTUs. Only OTUs containing ≥ 0.5% of the total abundances were presented, and bootstrap values over 50% based on 1000 replicates were shown. L, larval; LJ, large juvenile; SJ, small juvenile.

Although we identified some specific species according to the Silva database of bacteria and FunGene database (http://fungene.cme.msu.edu/) of SRB and SOB, about 58% OTUs of bacteria, 80% OTUs of SRB and 20% OTUs of SOB were unclassified. We further used the neighbour‐joining method to identify the phylogenetic position of those unidentified OTUs according to the sequence similarity. In particular, we picked up the OTUs with their abundance over 0.5% of 16S rRNA, *dsrB* and *soxB* gene sequences, then constructed the phylogenetic tree with 21, 40 and 34 OTUs remained for 16S rRNA, *dsrB* and *soxB* genes respectively (Fig. 3). For bacteria, most OTUs failed to annotate to the species level, and most of them belonged to different orders. In addition, these dominant bacteria were more abundant in large juvenile fish pond sediments. For SRB, only OTU_52 (*Desulfobulbus propionicus*), OTU_62 (*Desulfobulbus propionicus*) and OTU_449 (bacterium enrichment culture clone S2S‐F7) were well matched to the SRB database. OTU_445 and OTU_175 were abundant in all types of sediments, and they appeared to be closely related to *Desulfovibrionales*. Also, we found that a novel *dsrB* cluster (including OTU_1136, OTU_1137, OTU_1140, OTU_1141, OTU_1143, OTU_1149 and OTU_1175) was more abundant in larval and small juvenile fish ponds compared to large juvenile fish ponds. For SOB, 27 OTUs were annotated. *Dechloromonas aromatica,* OTU_163 (closely related to *Acidithiobacillales*), *Thiobacillus denitrificans*, *Halochromatium glycolicum* and *Leptothrix cholodnii* were abundant in all types of sediments.

### Sulfur compound accumulation and sediment suspension shaped the microbial communities in aquaculture pond sediments

**Fig. 4:**
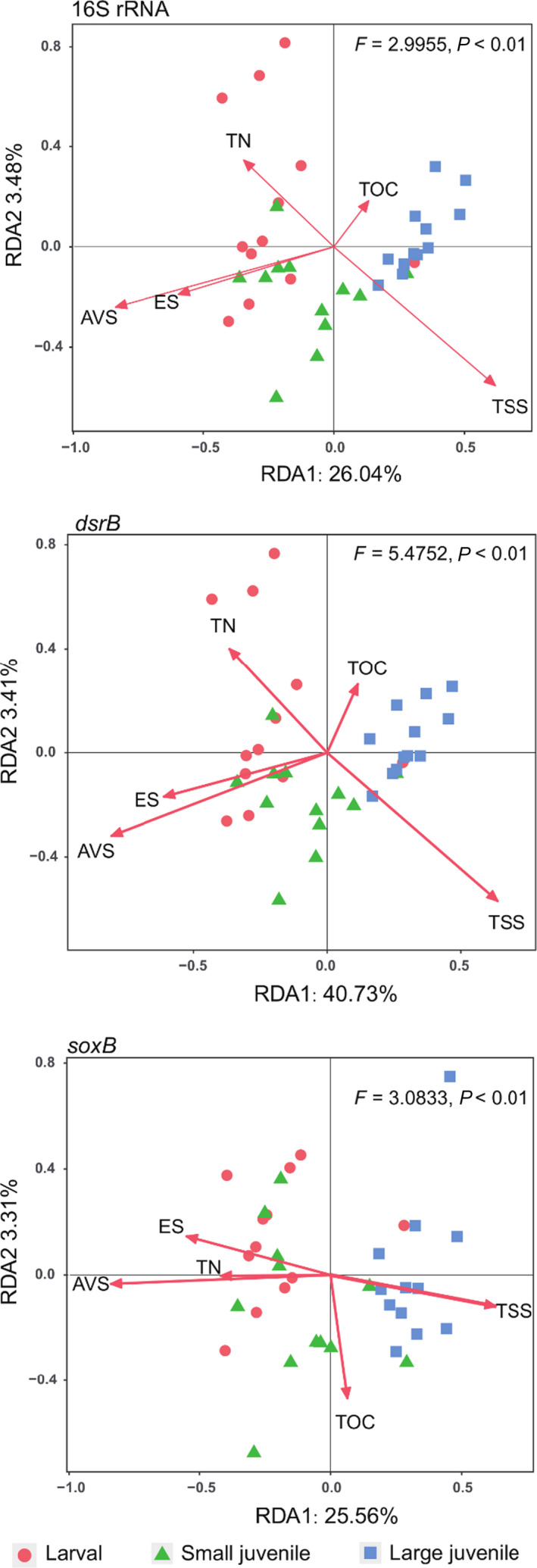
Redundancy analysis (RDA) ordination showing the relationships between bacterial communities (16S rRNA)/sulfate‐reducing bacteria (*dsrB*)/sulfur‐oxidizing bacteria (*soxB*) and environmental factors. AVS, acid‐volatile sulfur; ES, elemental sulfur; TN, total nitrogen; TOC, total organic carbon; TSS, total suspended solids.

**Fig. 5:**
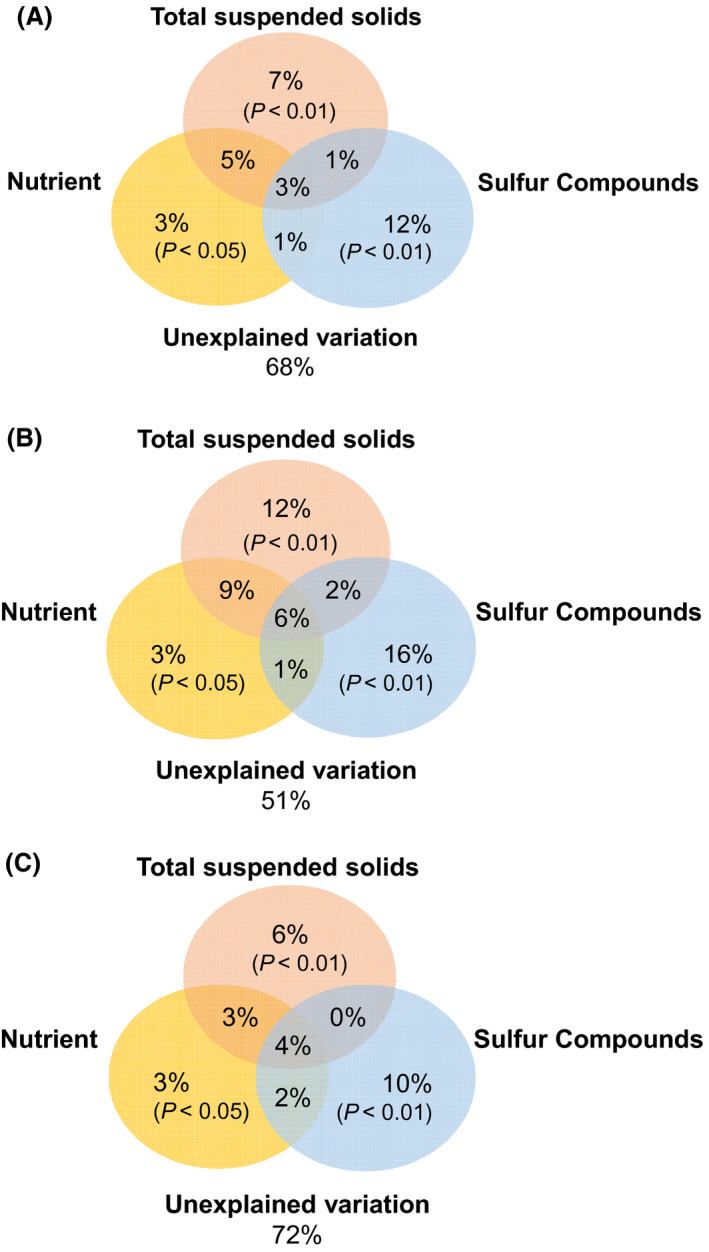
Variation partition analysis (VPA) showing the effects of environmental factors on the bacterial communities (A), sulfate‐reducing bacteria (B) and sulfur‐oxidizing bacteria (C) in sediments. Nutrient loading including total nitrogen (TN) and total organic carbon (TOC); sulfur compounds including total sulfur (TS), acid‐volatile sulfur (AVS) and elemental sulfur (ES); sediment suspension reflected by total suspended solids (TSS).

RDA analysis was used to find key factors driving microbial community variations across the three different types of aquaculture pond sediments. Several environmental variables were removed by forward‐selection algorithm in order to develop a robust model (Fig. 4). Final RDA showed that TOC and TN explained few variations in community structure of bacteria, SRB and SOB (*P > *0.1). While TSS significantly affected the distribution of bacterial communities (*F* = 3.00, *P = *0.01), SRB (*F* = 5.50, *P = *0.003), and SOB (*F* = 2.30, *P = *0.03). The AVS was another most important environmental factor that affected the distribution of bacterial communities (*F* = 8.96, *P = *0.001), SRB (*F* = 16.30, *P* = 0.001) and SOB (*F* = 9.19, *P* = 0.001). More specifically, TSS was strongly associated with microbial communities in large juvenile fish ponds, whereas ES and AVS were closely correlated with microbial communities of larval and small juvenile fish ponds. Mantel tests further verified that TS, AVS, ES and TSS were significantly correlated with the community structure of bacteria SRB and SOB (*P* < 0.05), and TOC and TN were only correlated with the community structure of SOB (Fig. S3). To better quantify the relative contributions of subgroups of environmental parameters to the variations in community structure of bacteria, SRB and SOB (at OTU level), we conducted a variance partitioning analysis (VPA) on three subgroups of factors (i.e. nutrient loading, sulfur compounds, sediment suspension). Overall, sulfur compounds and sediment suspension explained more variations than nutrient loading for variations in community structure of bacteria (Fig. 5A), SRB (Fig. 5B) and SOB (Fig. 5C).

Furthermore, linear regression was used to examine how sediment suspension and sulfur compounds influenced abundances of SRB and SOB. Results indicated that abundances of SRB and SOB increased with increasing of sediment suspension, whereas the abundance of SRB decreased with increasing of sulfur compounds (Fig. S4).

## Discussion

Sulfur cycling mediated by SRB and SOB plays an important role in sediments of aquaculture ecosystems. We found that aquaculture ponds cultured with large juvenile fish (grass carp) showed lower content of sulfur compounds but higher sediment suspension, which resulted in higher abundances of SRB and SOB and different microbial community structures in sediments compared to ponds cultured with larval fish.

The abundances and structures of sulfur‐cycling microbial communities differed among ponds cultured with different sized fish, which could be due to environmental discrepancies between pond sediments. AVS pool in sediments is highly dynamic (Morse and Rickard, 2004) and varies with sediment‐type, especially in high sulfidation environments (Rickard and Morse, 2005). However, concentration of AVS in sediments depends on the sulfate reduction rate of SRB (Liu *et al*., 2010). In this study, alpha diversity indexes and qPCR results all showed the ponds with large juvenile fish harboured more abundant SRB compared to those for larval fish ponds. According to the environmental standards of AVS for evaluating aquatic systems (Japan Fisheries Resource Conservation Association, 1995), ponds with larval and small juvenile fish were classified as ‘moderately polluted’, whereas ponds with large juvenile fish could be ‘unpolluted’. Theoretically, SRB should be more abundant in larval and small juvenile fish ponds due to high concentrations of AVS. Meanwhile, sulfate concentration showed no significant (*P *> 0.05) differences among ponds, and that was also not significantly correlated with microbial communities. Therefore, sulfate may not be the primary substrate to produce AVS in these aquaculture ponds. For instance, some volatile sulfur compounds can be produced from sulfur‐containing amino acids by methanogens and SRB, which is rich in formulated feeds (Holmer and Storkholm, 2001; Lu *et al*., 2013; Sun *et al*., 2015). Beyond that, AVS may be derived from soluble sulfide (Morse *et al*., 1987). Thus, the sulfur‐containing organic matters may be an important source of AVS except for sulfate in aquaculture pond sediments. However, this conclusion still needs further experiments to confirm.

The microbial community in farming sediments could be affected by organic enrichment (White, 2003). However, a previous study demonstrated that marine sediments with different levels of organic enrichments showed no prominent differences in SRB (Yoshida* et al*. , 1982). Our Mantel tests and RDA ordination showed that TOC and TN only weakly correlated with community structures of SRB and SOB, which indicated that accumulation of uneaten feeding food had little correlation with sulfur‐cycling microorganisms in aquaculture sediments. There may have two reasons. First, fish disturbance to sediment can inhibit organic matter accumulation (Brummett, 2000). Second, large fish ponds have more feed input but high utilization efficiency (Einen and Roem, 1997). All of these can reduce the difference of organic matter depositions in sediments among different aquaculture ponds. Instead, sulfide generated from microbial metabolism would influence the microbial activity in sediments (Visscher *et al*., 1995; Lyimo *et al*., 2009). We found that sulfur compounds showed strong correlations with microbial communities via RDA plot and VPA analysis, especially SRB and SOB. Moreover, linear regression indicated that high levels of sulfur compounds led to low abundance of SRB, as some SRB species could be restrained by sulfide at high concentrations (Reis *et al*., 1992). Sulfide also can inhibit SRB even at a low concentration (Takahashi *et al*., 2008). Thus, sulfur compounds like TS and AVS were more important than TOC and TN in influencing the community structure of SRB. High concentrations of sulfur compounds in aquaculture pond sediments would inhibit the abundance of SRB.

Disturbance to sediments by grass carp reshaped microbial communities in sediments. Since aquaculture ponds are closed systems and usually have no obvious disturbances to sediments expect fish activities. Animal disturbance to water–sediment interfaces frequently happened in ponds and lakes (Kristensen *et al*., 2012), and fish activities could increase the water turbidity (Adamek and Marsalek, 2013). Furthermore, bioturbation would accelerate the release of nutrients (like total nitrogen) into overlying water (Hansen *et al*., 1998), the suspended particles could also promote nitrification in the water (Xia *et al*., 2004). In this study, TSS, TN and nitrate contents in the water were significantly higher in large juvenile fish ponds, indicating strong disturbances caused by larger grass carps. The RDA and VPA results also showed the strong disturbance caused by fish would affect the distribution of SRB and SOB in aquaculture ponds. Meysman *et al*. (2005) found that the rate of sulfate reduction enhanced with increasing perturbation. Our linear regression results also showed that sediment suspension caused by fish activities increased the abundance of SRB and SOB. Fish disturbance disturbed the anoxic microenvironment and oxidized the chemical compounds through sulfide oxidation (Bertics and Ziebis, 2009), thus extended micro‐niches into an oxidized zone (Zorn *et al*., 2006). Although SRB are generally described as obligate anaerobes, some groups like *Desulfovibrio* possess a complex aggregation of enzymes to defence against oxidative stress (Dolla *et al*., 2006), and some SRB even could use O_2_ as a terminal electron acceptor for their aerobic growth (Lefèvre *et al*., 2016). A previous study found that disturbance could increase SRB abundance even in an aerobic environment (Bertics and Ziebis, 2009). In addition, SOB can use oxygen as an oxidizing agent during the oxidation of sulfide (Pokorna and Zabranska, 2015), which could be facilitated by fish disturbance. Furthermore, SOB can transform hazardous sulfur compounds and serve as bioremediation strategies (Krishnani *et al*., 2010a). Our results indicated that fish disturbance increased the abundance of SRB and SOB in upper sediment layers, further enhanced the sulfate reduction and sulfur oxidation processes.

**Fig. 6:**
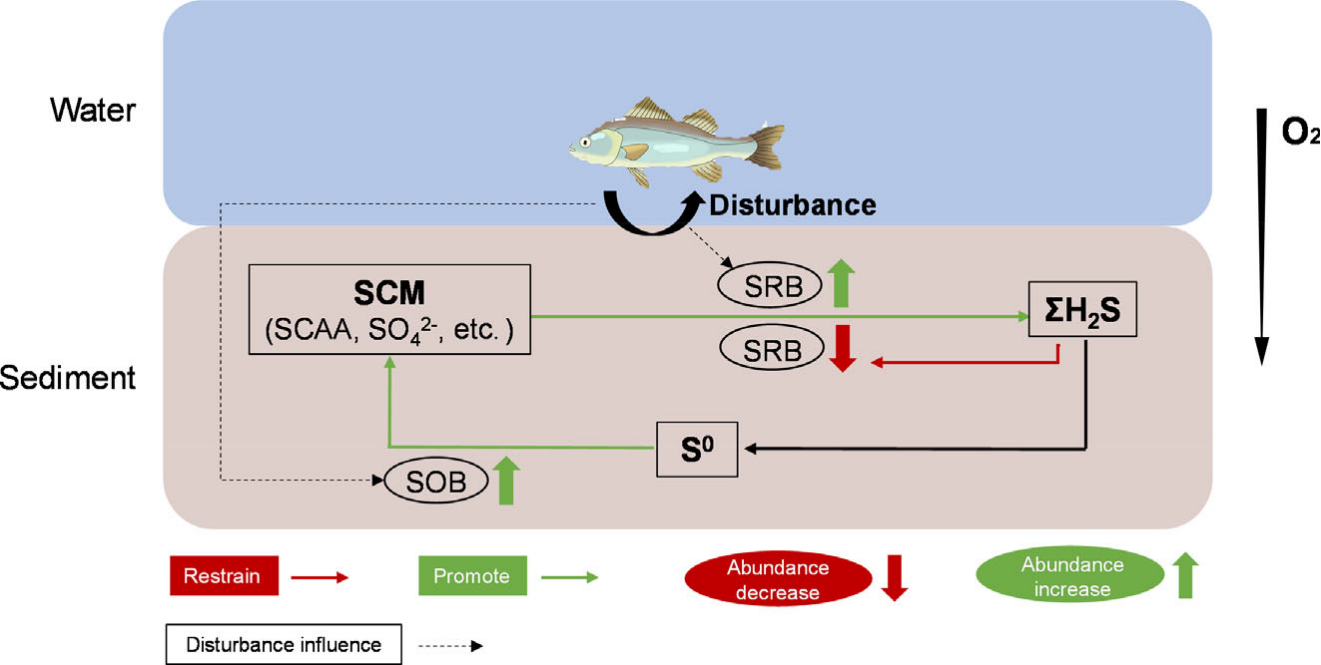
Putative model showing the sulfur cycling dominated by sulfate‐reducing bacteria (SRB) and sulfur‐oxidizing bacteria (SOB), and their major influence factors in aquaculture ponds. SCM, sulfur‐containing materials; SCAA, sulfur‐containing amino acids.

Based on findings of this study and our current knowledge, we proposed a putative model of sulfur cycling dominated by SRB and SOB in aquaculture ponds (Fig. 6). SRB transformed sulfur‐containing materials (SCM) into sulfide through sulfate reduction process, and most sulfides were oxidized to sulfate through sulfur oxidation by SOB. The sulfate reduction process was affected by sulfur compounds in sediments, and a high concentration of sulfur compounds would inhibit SRB activity. Meanwhile, sulfur cycling mediated by SRB and SOB would be facilitated by the fish disturbance to sediments, thus activated the sulfur cycling.

## Conclusions

We found that the abundance of SRB and SOB and microbial community structure were significantly different in aquaculture ponds cultured with different sizes of grass carp. In aquaculture pond sediments, SRB produced AVS through sulfate reduction, and SOB was especially important to reduce AVS. High concentrations of sulfur compounds (TS, AVS and ES) could inhibit SRB and sulfate reduction. Disturbance to sediments by fish activities could enhance the sulfate reduction and sulfur oxidation in sediments. These findings help us to better understand microbial sulfur cycling in aquaculture ponds and provide new insights into microbial manipulation of healthy aquaculture environment.

## Experimental procedures

### Study area and sampling procedures

Samples were collected from grass carp aquaculture ponds at Nansha (22°60′97.88″N, 113°62′18.05″E), Guangdong province of China on 18 April and 31 May 2018. Three kinds of ponds cultured with different sizes of grass carps, i.e., larval fish (L), small juvenile fish (SJ) and large juvenile fish (LJ) were sampled. Each pond is 1.5 km^2^ in area, and about 2.0 m in‐depth without water exchanging during our sampling period. In all ponds, the clays were removed and the sediments were further treated with quicklime before culturing grass carp. These ponds used for culture already lasted for nearly one year before sampling. During the one year of culturing, each pond only used for culturing one sized fish, and the fish size grew to big enough would be transferred to another corresponding type of ponds. On the first sampling time, the culture of larval fish had just begun three days ago, and the culture of small and large juvenile fish had lasted for half a month. The benthic organisms were not common in the pond sediment. During 18 April and 31 May, in larval fish ponds, the fish initially weighted ~ 0.2 g and increased by 0.8 g, the density was 750 fish per m^2^; in small juvenile fish ponds, the fish initially weighted ~ 50 g and increased by 150 g, the density was 30 fish per m^2^; in large juvenile fish ponds, the fish initially weighted ~ 400 g and increased by 180 g, the density was 5 fish per m^2^. The feed supply is commercial crumbled (larval fish) or pelleted (juvenile fish) formulated feed with a ratio of 5% body weight per day. For each size of grass carp, three ponds were sampled as biological templates, and three surface water (1 l each) and three sediment (500 g each) samples were collected from the left, centre and right of each pond. Then, mixed three sediment samples and three water samples in each pond for the sampling occasion of 18 April; however, the sampling occasion of 31 May was not mixed, and thus, totally there were 36 water samples and 36 sediment samples for further analysis. The water (about 50 cm below water level) and upper sediments (0–8 cm) were obtained by using 5‐litre hydrophore sampler and Van Veen Grab Sampler respectively. All samples were kept in ice box and brought back to the laboratory. A subset of ~ 50 g of each sediment sample was separated and frozen at −80°C for further DNA extraction, and the rest of sediments and water samples were used for physicochemical parameter tests.

### Physicochemical analyses

A subset of 10 g of each sediment sample was separated for measuring total organic carbon (TOC), total nitrogen (TN) and total sulfur (TS). Specifically, sediment was oven‐dried at 65 °C till a constant weight and sieved through a 150‐μm sieve. Then, 200 mg dry sediment of each sample was mixed thoroughly with 1 ml of 0.5 mol l^−1^ hydrochloric acid and then dried at 80°C for 4–5 h. TOC was examined by using a PRIMACS^MCS^ TOC Analyzer (Skalar, Netherlands). For measuring TN and TS, about 6 mg dry sediment of each sample was loaded into a CHNS/O Elemental analyser (Vario EL cube, Germany). Total suspended solids (TSS) were measured using a modified method to avoid plankton influence in the water, and the TSS were used to represent the degree of sediment suspension. Specifically, a glass fibre filter with pore size of 0.45 μm was dried at 400°C for 4 h and weighed as a blank control, 200 ml water sample was filtered through such filter was dried at 400°C for 4 h and weighed, and TSS was calculated from their subtraction. Transparency was measured by using a Secchi disk. Total nitrogen of water was analysed according to the method as described by Parsons and Takahashi (1973), and nitrate was analysed by using the method described previously (Ebina *et al*., 1983).

Fresh sediments were used to measure pH, sulfate (SO_4_^2−^), acid‐volatile sulfide (AVS) and elemental sulfur (ES). Briefly, pH was determined by using a pH electrode (SevenCompact™ pH Meter S210; Mettler Toledo, Swiss) (Ihara *et al*., 2017). For analysing the SO_4_^2−^, about 20 g wet sediments were centrifuged in a tube at 4000 × *g* for 10 min to obtain pore water. The pore water was filtered through H‐pretreatment columns and C18‐pretreatment columns (ANPEL, Shanghai) to remove metal ion and organic matters respectively. Then, SO_4_^2−^ was measured by ion chromatography (Thermo Fisher, ICS‐600). AVS measured here included all sulfide that can release H_2_S if treated with acid (e.g. H_2_S, HS^−^, FeS) (Rickard and Morse, 2005). For measuring the AVS, 6 g fresh sediments were transferred into 250‐ml polyethylene bottle with a small vial containing 10 ml 3% alkaline zinc solution. After purging with nitrogen gas for 5 min to remove oxygen, 10 ml 9 M HCl was injected, then closed syringe stopcock of the polyethylene bottle immediately (Brouwer and Murphy, 2010). An additional 1 ml 1 M ascorbic acid was injected into the bottle to avoid interference of ferric ions. After diffusion and reaction at room temperature for 4 h, sulfide precipitates in the alkaline zinc solution were collected and measured by iodometric titration method (Hsieh and Yang, 1989; Hsieh and Shieh, 1997). ES was defined as the sulfur extracted with methanol from sediment samples and measured by Reversed‐Phase HPLC. About 1 g fresh sediment was transferred into 50‐mL polyethylene centrifuge tube with 25 ml pure methanol, and shook for 24 h in a rotary shaker (25°C, 160 r min^−1^). After centrifuged (3000 rpm min^−1^) for 5 min, the supernatant was filtered through 0.22‐μm membrane and analysed by HPLC using a C18 reversed‐phase column (Thermo, Eclipse XDB‐C18 5 μm, 150 × 4.6 mm I.D). The eluent was composed of ratio at methanol/water = 95:5 (v/v) with a flow rate of 0.8 ml min^−1^, and a UV detection performed at 254 nm wavelengths to quantify elemental sulfur (Zopfi *et al*., 2004; Knossow *et al*., 2015).

### DNA extraction and PCR amplification

Only sediment samples were involved in microbial analysis. Total microbial DNA was extracted by using a freeze‐grinding method with sodium dodecyl sulfate (SDS) (Zhou *et al*., 1996), followed by PowerSoil DNA Kit (Mo Bio Laboratories Inc., Carlsbad, CA, USA) according to the manufacturer’s instructions. The concentration and quality of extracted DNA were determined by using NanoDrop One spectrophotometer (Thermo Fisher Scientific, MA, USA), diluted to the same concentration (10 ng μl^−1^) and stored at −80°C until subsequent analyses.

The V4 region of the 16S rRNA gene, *dsrB* and *soxB* genes was amplified by primer sets used previously (Table S1). Amplicons for Illumina sequencing were prepared following the barcoded PCR procedures: 50 µl reaction system including 10 µl buffer (5×), 0.2 µl Q5 High‐Fidelity DNA Polymerase, 10 µl High GC Enhancer, 1 µl dNTP, 10 µM of each primer and 60 ng genomic DNA. The amplification of 16S rRNA gene was conducted with an initial denaturation at 95°C for 5 min, followed by 30 cycles of 95°C for 30 s, 53°C for 45 s and 72°C for 1 min, and a final extension at 72°C for 5 min. PCR cycling procedures for the *dsrB* gene are 30 cycles of 95°C for 30 s, 50°C for 30 s and 72°C for 30 s; for the *soxB* gene are 30 cycles of 95°C for 30 s, 55°C for 30 s and 72°C for 40 s. The positive PCR products were purified through VAHTSTM DNA Clean Beads.

A second round Solexa PCR was performed in a 40 µl reaction which contained 20 µl (2×) Phµsion HF MM, 8 µl ddH_2_O, 10 µM of each primer and 10 µl PCR products from the first step. Thermal cycling conditions were as follows: an initial denaturation at 98°C for 30 s, followed by 10 cycles at 98°C for 10 s, 65°C for 30 s and 72°C for 30 s, with a final extension at 72°C for 5 min. PCR products were visualized using 1.8% agarose gels to obtain target band, purified as the previous step, then quantified by Quant‐iT™dsDNA HS Reagent and mixed fully. All mixtures were added to a 1.8% agarose gel, the gel electrophoresis was performed with 120 V for 40 min, and the specific band was excised and purified.

### High‐throughput sequencing and data processing

High‐throughput sequencing was performed on Illumina platforms in the Biomarker Technologies Corporation, Beijing. Specifically, fragments of the 16S rRNA gene (291 bp, V4 region) and *dsrB* gene (350 bp) were sequenced by Illumina HiSeq PE250 platform, and the *soxB* gene (470 bp) was sequenced by Illumina MiSeq PE300 platform.

The obtained raw paired‐end 16S rRNA, *dsrB* and *soxB* gene sequences were filtered ambiguous base(s) (“N”). After trimming and identifying sequencing regions, the obtained high‐quality fragments were aligned in Quantitative Insights Into Microbial Ecology (QIIME) v1.9.1 (http://qiime.org/). The aligned 16S rRNA gene sequences without chimeras were compared based on the Ribosomal Database Project (RDP) database by using USEARCH v11 (Edgar, 2013). The operational taxonomic units’ (OTUs) table was generated by using UNOISE method with 97% cut‐off (Edgar, 2016). OTUs were grouped into different taxonomy by using Silva database release 132. The frameshifts of the *dsrB* and *soxB* genes were corrected used the RDP FrameBot tool in Galaxy pipeline (http://mem.rcees.ac.cn:8080/), and only reads whose translated proteins mapped to reference protein sequences were retained (Wang *et al*., 2013). After removing chimeras, the high‐quality sequences of functional genes were clustered into OTUs at a 90% identity threshold by QIIME and VSEARCH (Pelikan *et al*., 2015). The reference database for annotation was downloaded from FunGene Pipeline (http://fungene.cme.msu.edu/), and the comparison identity for taxonomy assignments was 0.9 and 0.7 for the *dsrB* and *soxB* respectively. Raw OTUs obtained from the *dsrB* and *soxB* gene sequences were filtered with an average relative abundance of lower than 1/10,000. Afterwards, microbial alpha‐diversity (observed OTUs and Shannon) was calculated by QIIME (Caporaso *et al*., 2010). A phylogenetic tree was constructed in MegaX and iTOL (https://itol.embl.de/) using a neighbour‐joining algorithm with 1000 bootstrap (Kumar *et al*., 2018).

### Quantitative real‐time PCR analysis (qPCR)

The abundance of 16S rRNA, *dsrB* and *soxB* genes was profiled by qPCR. All PCR products were purified by using DiaSpin DNA Gel Kit (Sangon, Shanghai, China), and cleaned targeted gene fragments were extracted from Trans1‐T1 using pEASY^®^‐T1 Simple Cloning Kit (TransGen, Beijing, China). Plasmids were sequenced and verified using the basic local alignment search tool (BLAST) (http://blast.ncbi.nlm.nih.gov/Blast.cgi). The concentrations of plasmids containing target sequences were measured by Qubit™ Assays fluorometer (Thermo Fisher Scientific). The initial stand curves were diluted by gradient starting from 10‐fold dilutions of plasmids carrying the targeted genes.

The qPCR was performed using the primers used previously (Table S1) according to the methods as described by Jiang *et al*. (2015) and He *et al*. (2015). All qPCR experiments were conducted on a BIO‐RAD CFX96™ Real‐Time System with SYBR Green method with triplicate replicates. The 12‐µl volume of qPCR mixture contained 6 µl iTaq™ Universal SYBR Green Supermix (BIO‐RAD), 0.3 µM each primer and 1.6 µl template DNA. The amplification procedure was as follows: initial denaturation for 5 min at 95°C, followed by 40 cycles of denaturation at 95°C for 15 s, annealing at 52°C for 30 s (16S rRNA), or at 60°C for 40 s (*dsrB*), or 56.3°C for 45 s at (*soxB*), and extension at 72°C for 45 s. The data were analysed using the CFX Maestro software, and all correlation coefficients (*R* ^2^) of standard curves were > 0.99. The amplification efficiencies (E) of 16S rRNA, *dsrB* and *soxB* genes were 110%, 85.7% and 93.4% respectively.

### Statistical analysis

Microbial community dissimilarities were visualized by principal coordinate analysis (PCoA) based on Bray–Curtis distance using vegan package (Yao *et al*., 2014). Pearson’s correlation coefficient was used to determine the relationship between environmental factors. The relationships between microbial communities and environmental factors were determined by the Mantel tests and redundancy analysis (RDA) using vegan package. The statistical significance of the RDA model was tested by Monte Carlo permutation tests (with 999 permutations). Variation partitioning analysis (VPA) was performed to quantify the relative contributions of environmental variables using the varpart procedure in the vegan package. All the analyses as described above were performed using the R statistical programs v3.4.4. Linear correlation of bacterial abundance and environmental variances were conducted using software graphpad prism 7. Differences of environmental factors and taxonomy between different ponds were performed by one‐Way ANOVA and Tukey’s multiple comparison test in graphpad prism 7. The significant level of all data was set at *P < *0.05.

## Conflict of interest

All authors declare no conflict of interest. None of the materials presented in this manuscript has been previously published, nor are they under consideration for publication elsewhere.

## Finding information

This work was supported by the National Natural Science Foundation of China (31672262, 31802350, 31800417), the Fundamental Research Funds for the Central Universities (19lgzd28, 19lgpy164), the Hundred Talents Program through Sun Yat‐sen University (38000‐18821107) and the Foundation of the State Key Laboratory of Applied Microbiology Southern China (SKLAM005‐2018).

## Supporting information


**Table S1.** Primers used for amplification of 16S rRNA, *dsrB* and *soxB* genes.
**Fig. S1.** Microbial richness and diversity in sediments of aquaculture ponds with different sizes of grass carp. Mean values were plotted with the standard deviations (*n *= 12). Significance (*P* < 0.05) was tested according to one‐way ANOVA, followed by Tukey’ s multiple comparison test. The presence of different letters denoted significant differences, and the same letter indicated no significant differences.
**Fig. S2.** Relative abundances of top 10 genera of bacterial communities (16S rRNA), sulfate‐reducing bacteria (*dsrB*) and sulfur‐oxidizing bacteria (*soxB*). L: larval; SJ: small juvenile; LJ: large juvenile.
**Fig. S3.** Mantel tests showing the relationships between microbial communities and environmental factors. The thickness of connecting lines represent correlation level, and wider lines indicate stronger correlation. The Pearson’s test showing the relationship between key environmental factors, only values matched |r| > 0.5 and *P* < 0.05 were retained, otherwise r value transformed to 0. TSS: total suspended solids; TS: total sulfur; AVS: acid‐volatile sulfur; ES: elemental sulfur; TOC: total organic carbon; TN: total nitrogen.
**Fig. S4.** Linear regression analysis showing the relationships between abundances of sulfate‐reducing bacteria (reflected by *dsrB* gene) or sulfur‐oxidizing bacteria (reflected by *soxB* gene) and key environmental factors. TSS: total suspended solids; TS: total sulfur; AVS: acid‐volatile sulfide.

## Data Availability

The raw sequencing data can be found at the National Centre for Biotechnology Information (NCBI) Sequence Read Archive (SRA) under accession numbers of SUB6464626 (16S rRNA), SUB6467726 (*dsrB*) and SUB6467851 (*soxB*).
