# Supplementary material for: Fish growth enhances microbial sulfur cycling in aquaculture pond sediments
Source: Microb Biotechnol. 2020 Jul 6;13(5):1597–610. doi: 10.1111/1751-7915.13622 (PMC7415356; doi:10.1111/1751-7915.13622)
Supplement: Supplementary file 1 — Table S1. Primers used for amplification of 16S rRNA, dsrB and soxB genes. Fig. S1. Microbial richness and diversity in sediments of aquaculture ponds with different sizes of grass carp. Mean values were plotted with the standard deviations (n = 12). Significance (P < 0.05) was tested according to one‐way ANOVA, followed by Tukey’ s multiple comparison test. The presence of different letters denoted significant differences, and the same letter indicated no significant differences. Fig. S2. Relative abundances of top 10 genera of bacterial communities (16S rRNA), sulfate‐reducing bacteria (dsrB) and sulfur‐oxidizing bacteria (soxB). L: larval; SJ: small juvenile; LJ: large juvenile. Fig. S3. Mantel tests showing the relationships between microbial communities and environmental factors. The thickness of connecting lines represent correlation level, and wider lines indicate stronger correlation. The Pearson’s test showing the relationship between key environmental factors, only values matched |r| > 0.5 and P < 0.05 were retained, otherwise r value transformed to 0. TSS: total suspended solids; TS: total sulfur; AVS: acid‐volatile sulfur; ES: elemental sulfur; TOC: total organic carbon; TN: total nitrogen. Fig. S4. Linear regression analysis showing the relationships between abundances of sulfate‐reducing bacteria (reflected by dsrB gene) or sulfur‐oxidizing bacteria (reflected by soxB gene) and key environmental factors. TSS: total suspended solids; TS: total sulfur; AVS: acid‐volatile sulfide. [file MBT2-13-1597-s001.docx]

**Supplementary data**

**Table S1** Primers used for amplification of 16S rRNA, *dsrB* and *soxB* genes.

| Primer | Gene | Function | Primer sequence (5′-3′) | Reference |
| --- | --- | --- | --- | --- |
| 515F | 16S rRNA | High-throughput sequencing; qPCR | GTGYCAGCMGCCGCGGTAA | (Tian et al., 2017) |
| 806R | 16S rRNA | High-throughput sequencing; qPCR | GGACTACNVGGGTWTCTAAT | (Tian et al., 2017) |
| DSRp2060-F | *dsrB* | Functional gene sequencing; qPCR | CAACATCGTYCAYACCCAGGG | (Müller et al., 2015) |
| DSR4-R | *dsrB* | Functional gene sequencing; qPCR | GTGTAGCAGTTACCGCA | (Müller et al., 2015) |
| soxB693-F | *soxB* | Functional gene sequencing; qPCR | ATCGGNCARGCNTTYCCNTA | (Geets et al., 2006) |
| soxB1164B-R | *soxB* | Functional gene sequencing | AARTTNCCNCGNCGRTA | (Birte et al., 2010) |
| soxB1164BK145-R | *soxB* | qPCR | AAGTTGCCDCGNCGRTA | (Luo et al., 2018) |

**Reference**

Birte, M., Imhoff, J.F., and Jan, K. (2010) Molecular analysis of the distribution and phylogeny of the *soxB* gene among sulfur-oxidizing bacteria - evolution of the Sox sulfur oxidation enzyme system. *Environ Microbiol* **9:** 2957-2977.

Geets, J., Borremans, B., Diels, L., Springael, D., Vangronsveld, J., Lelie, D.V.D., *et al.* (2006) *DsrB* gene-based DGGE for community and diversity surveys of sulfate-reducing bacteria. *J Microbiol Methods* **66:** 194-205.

Luo, J., Tan, X., Liu, K., and Lin, W. (2018) Survey of sulfur-oxidizing bacterial community in the Pearl River water using *soxB* , *sqr* , and *dsrA* as molecular biomarkers. *Biotech* **8:** 73.

Müller, A.L., Kjeldsen, K.U., Rattei, T., Pester, M., and Loy, A. (2015) Phylogenetic and environmental diversity of DsrAB-type dissimilatory (bi)sulfite reductases. *ISME J* **9:** 1152-1165.

Tian, H., Gao, P., Chen, Z., Li, Y., Li, Y., Wang, Y., *et al.* (2017) Compositions and abundances of sulfate-reducing and sulfur-oxidizing microorganisms in water-flooded petroleum reservoirs with different temperatures in China. *Front Microbiol* **8:** 143-156.


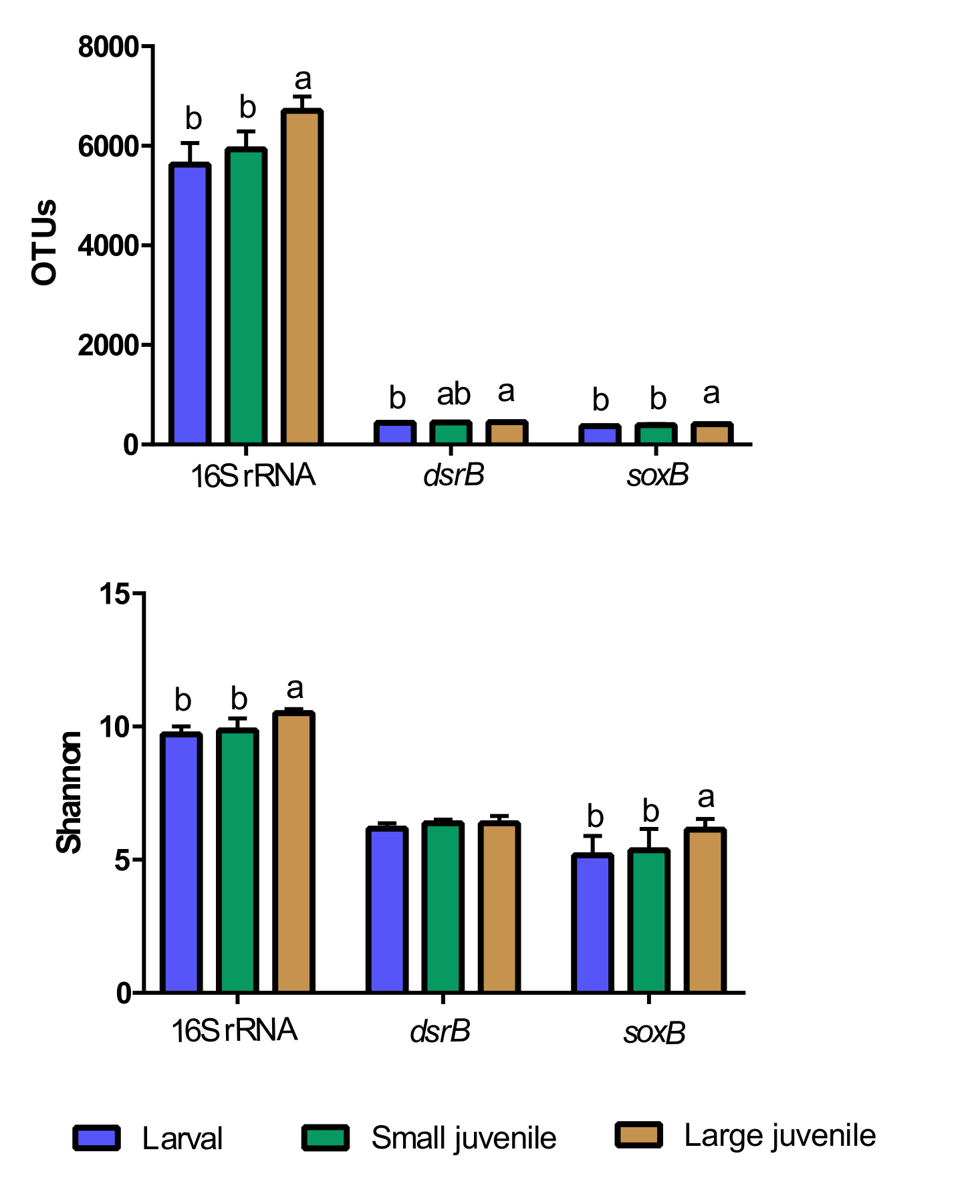


**Fig. S1.** Microbial richness and diversity in sediments of aquaculture ponds with different sizes of grass carp. Mean values were plotted with the standard deviations (*n*=12). Significance (*p* < 0.05) was tested according to one-way ANOVA, followed by Tukey’ s multiple comparison test. The presence of different letters denoted significant differences, and the same letter indicated no significant differences.


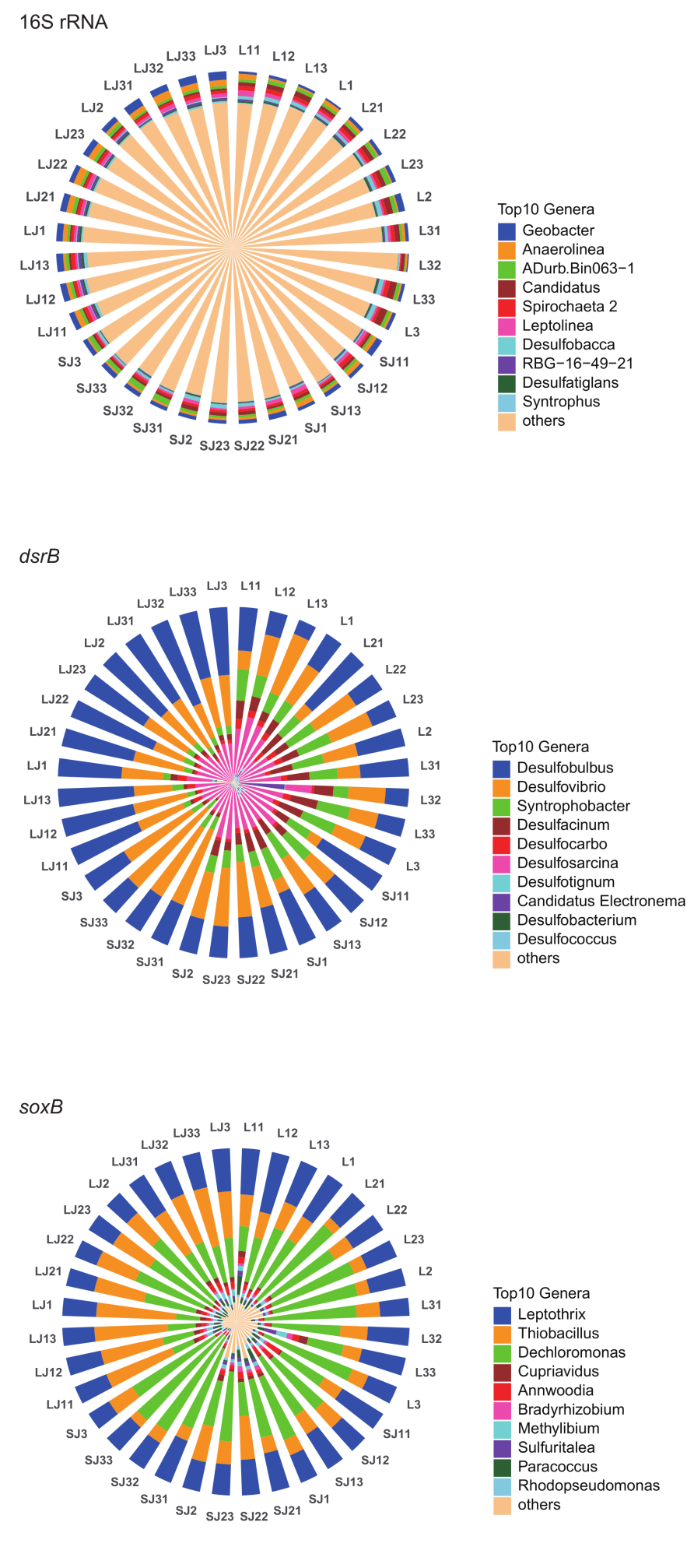


**Fig. S2.** Relative abundances of top 10 genera of bacterial communities (16S rRNA), sulfate-reducing bacteria (*dsrB*) and sulfur-oxidizing bacteria (*soxB*). L: larval; SJ: small juvenile; LJ: large juvenile.


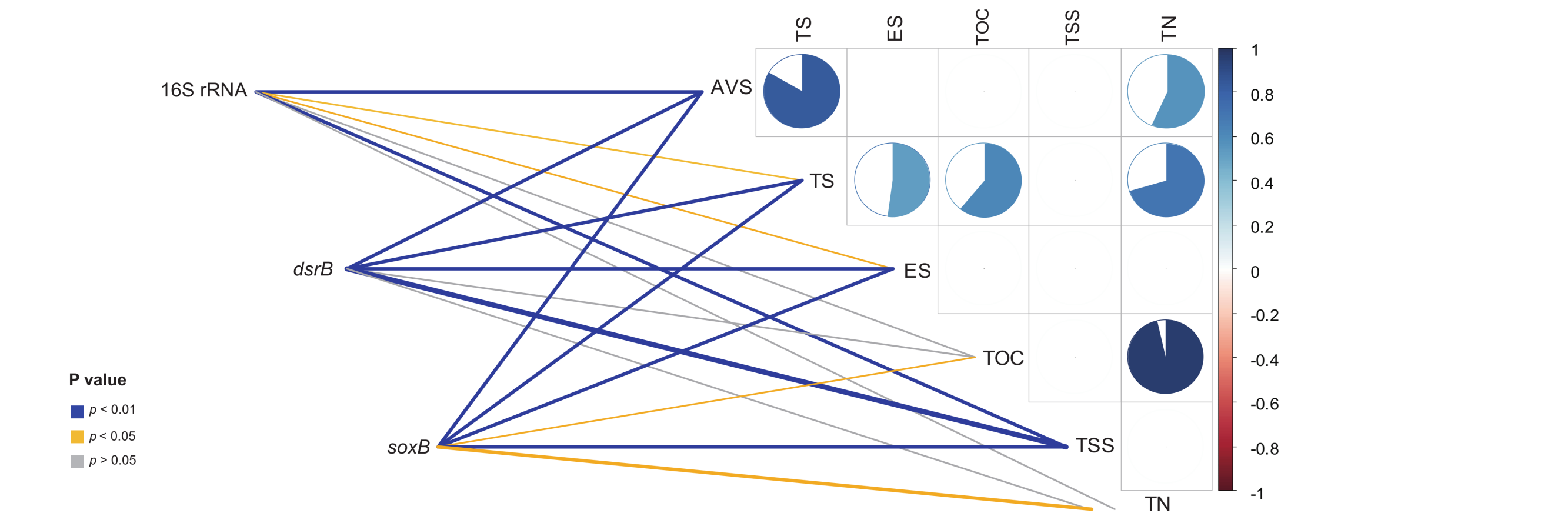


**Fig. S3.** Mantel tests showing the relationships between microbial communities and environmental factors. The thickness of connecting lines represent correlation level, and wider lines indicate stronger correlation. The Pearson’s test showing the relationship between key environmental factors, only values matched |r| > 0.5 and *p* < 0.05 were retained, otherwise r value transformed to 0. TSS: total suspended solids; TS: total sulfur; AVS: acid-volatile sulfur; ES: elemental sulfur; TOC: total organic carbon; TN: total nitrogen.


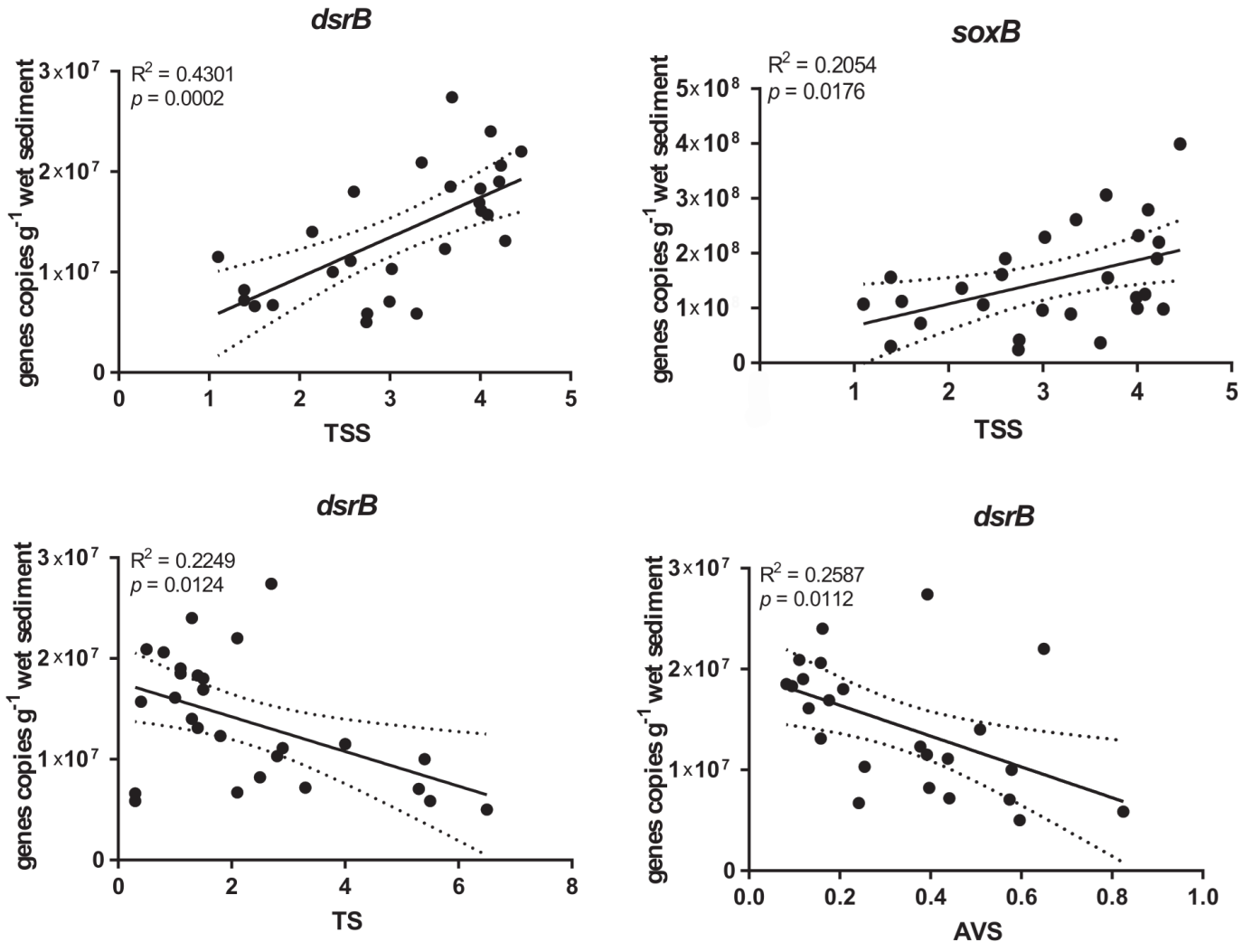


**Fig. S4.** Linear regression analysis showing the relationships between abundances of sulfate-reducing bacteria (reflected by *dsrB* gene) or sulfur-oxidizing bacteria (reflected by *soxB* gene) and key environmental factors. TSS: total suspended solids; TS: total sulfur; AVS: acid-volatile sulfide.
